# Management of the Axilla in Older Patients with Breast Cancer: Reassessing the Role of Sentinel Lymph Node Biopsy

**DOI:** 10.3390/cancers17172758

**Published:** 2025-08-24

**Authors:** Francisco Castillejos Ibáñez, Ernesto Muñoz Sornosa, Vicente López Flor, Marcos Adrianzén Vargas, María Teresa Martínez Martínez, Elvira Buch Villa

**Affiliations:** 1 Breast Unit, Department of General and Digestive Surgery, Hospital Clinico Universitario of Valencia, Department of Surgery, University of Valencia, Biomedical Research Institute, INCLIVA. Av. de Blasco Ibáñez, 17, 46010 Valencia, Spain; munoz.sornosa@gmail.com (E.M.S.); vlopezflor@yahoo.es (V.L.F.); marcos.adrianzen@gmail.com (M.A.V.); nineta03@gmail.com (E.B.V.); 2 Department of Medical Oncology, Hospital Clinico Universitario of Valencia, Department of Medicine, University of Valencia, Biomedical Research Institute, INCLIVA. Av. de Blasco Ibáñez, 17, 46010 Valencia, Spain; maitemartinez3@yahoo.es

**Keywords:** breast cancer, sentinel lymph node, elderly women, axillary staging, hormone receptor-positive, de-escalation

## Abstract

Background: Sentinel lymph node biopsy (SLNB) has traditionally been used to stage the axilla in early-stage breast cancer. However, its utility in women over 70 with hormone receptor-positive tumors and negative axillary imaging is increasingly questioned due to limited therapeutic benefit and potential complications. Objectives. To assess the feasibility of omitting SLNB in women aged 70 and older with clinically node-negative, luminal-type breast cancer. Methods: A retrospective analysis was conducted on women aged 70 and above with histologically confirmed invasive breast cancer, negative axillary imaging, and surgery between January 2021 and December 2024. Eligible patients were selected based on normal axillary ultrasound findings. All underwent SLNB. We examined demographics, clinical characteristics, surgical outcomes, and oncological variables such as recurrence and mortality. Results: A total of 149 women underwent surgery, with a mean age of 77.2 (5.24) years. SLNB was positive in 23.5% of cases, but only 6.7% required axillary dissection. Sensitivity and specificity of SLNB declined notably after age 76. No axillary or breast recurrences were reported. Most patients (89.9%) received hormonal therapy, while 11.4% had chemotherapy and 17.5% axillary radiotherapy. Outpatient management was feasible in 87.9% of cases, and no clinically significant lymphedema was observed. Conclusions: Omitting SLNB in women ≥70 years with luminal breast cancer and negative axillary imaging appears safe and does not compromise oncological outcomes. This strategy minimizes surgical risks and enhances quality of life, supporting a more tailored and less invasive approach to axillary management in older patients.

## Simple Summary

Sentinel lymph node biopsy is a common procedure used to check if breast cancer has spread to the lymph nodes. However, in women aged 70 and older with a specific type of early breast cancer and normal imaging of the underarm area, this procedure may not always be necessary. We studied 149 women over 70 who had surgery and found that skipping this test could be a safe option. Very few needed further treatment in the lymph nodes, and none had cancer return in the breast or underarm. Most women were treated successfully with hormone therapy and had a smooth recovery without serious complications. These findings suggest that older women with low-risk breast cancer might avoid this surgery without affecting their health. This could lead to less invasive care, fewer side effects, and better quality of life.

## 1. Introduction

Breast cancer (BC), whose incidence increases with age, is the most common malignancy in women, with more than 30% of diagnoses occurring in women over the age of 70 [1]. Sentinel lymph node biopsy (SLNB) has traditionally been the standard procedure for axillary staging in patients with early-stage BC, allowing the identification of regional lymph node involvement with lower morbidity than complete axillary lymph node dissection [2]. However, in recent years, the necessity of performing this procedure in elderly patients has been questioned due to its limited impact on overall survival and its constrained influence on therapeutic decision-making [3].

In this regard, recent studies have investigated the feasibility of omitting SLNB in women over 70 years of age with luminal-type BC and clinically and sonographically negative axillae at diagnosis. Consequently, the Choosing Wisely initiative recommends avoiding SLNB in these patients, as the information obtained does not significantly alter the therapeutic approach in the majority of cases [1]. Indeed, data from the NAFTA trial (North American Fareston versus Tamoxifen Adjuvant) trial [4], which included 1813 postmenopausal women with hormone receptor-positive invasive BC, and the International Breast Cancer Study Group Trial 10-93 [5], have shown that omitting axillary evaluation does not affect overall survival or locoregional recurrence in women with early-stage disease [3]. These findings have fueled ongoing debate about the necessity of SLNB in this patient subgroup [6].

Analysis of large cohort data and clinical trials has revealed that the rate of nodal involvement in patients with sonographically negative axillae ranges between 10% and 15% [3], suggesting that a significant proportion of women could avoid surgical intervention without compromising their prognosis. Furthermore, studies such as that by Marco Sanz et al. [7] have indicated that omitting SLNB in this patient group does not affect recurrence rates or overall survival, thereby supporting the use of axillary ultrasound as a primary staging tool, eliminating the need for intraoperative SLNB.

Despite these recommendations, more than 80% of women over the age of 70 continue to undergo SLNB [1]. Contributing factors include clinical inertia, concerns about potential undertreatment, and variability in risk perception among oncologists [1,3]. Nevertheless, evidence suggests that even when SLNB is omitted, the rate of subsequently detected nodal involvement remains low and does not substantially alter the requirement for adjuvant therapy [7]. Additionally, the impact on quality of life is significant, as omitting SLNB reduces the risk of lymphoedema, chronic pain, and arm dysfunction in elderly patients [6].

Against this background, our aim was to analyze our recent outcomes in patients over 70 years of age with breast cancer and a sonographically negative axilla, in order to evaluate the feasibility of omitting sentinel lymph node biopsy (SLNB) in this population. We hypothesized that, in the absence of suspicious findings on axillary ultrasound, SLNB may be safely avoided, potentially reducing surgical morbidity without compromising the oncologic management of breast cancer in older women.

This study seeks to contribute to the ongoing discussion regarding the optimization of BC management in elderly patients, by exploring strategies that minimize unnecessary interventions while preserving treatment effectiveness and enhancing quality of life.

## 2. Materials and Methods

### 2.1. Study Design and Setting

This was a retrospective study based on a prospectively maintained institutional database. It included all patients aged 70 years or older with a diagnosis of breast cancer (BC) who underwent surgery at our center between January 2021 and December 2024. All eligible patients who met the inclusion criteria during this period were included consecutively, without sampling.

### 2.2. Ethics Statement

This study was conducted in accordance with the ethical standards of the institutional research committee and the 1964 Helsinki Declaration and its later amendments. Moreover, the study protocol was approved by the Clinical Research Ethics Committee of the Hospital Clinico Universitario of Valencia during its regular meeting held on 10 July 2025 (Record No. 443). The approved protocol corresponds to version 1, dated 10 June 2025, with project number 2025/220. Informed consent was waived due to the retrospective nature of the study and the use of anonymized data, in accordance with institutional and national regulations.

### 2.3. Inclusion and Exclusion Criteria

#### 2.3.1. Inclusion Criteria:

- Age ≥ 70 years.
- Histologically confirmed invasive breast carcinoma.
- Negative axillary status confirmed by axillary ultrasound, magnetic resonance imaging (MRI), or positron emission tomography–computed tomography (PET–CT). Patients with suspicious imaging findings but negative preoperative biopsy (vacuum-assisted biopsy [VAB] or core needle biopsy [CNB]) were also included.
- No neoadjuvant treatment.
- Patients underwent either mastectomy or breast-conserving surgery.
- Sentinel lymph node biopsy (SLNB) performed.

#### 2.3.2. Exclusion Criteria:

- Patients who did not undergo surgical intervention.
- Absence of axillary assessment via SLNB.

### 2.4. Data Sources and Collection

Clinical data were retrospectively collected from the electronic medical records of our institution. All patient records are digitalized and stored in an integrated hospital information system. Data were extracted manually by the study team from medical charts, surgical reports, pathology records, and imaging studies. The institutional breast cancer registry, which is prospectively maintained, was used to identify eligible patients. All patients who met the inclusion criteria between January 2021 and December 2024 were included, with no sampling applied.

### 2.5. Preoperative Assessment and Surgical Management

All patients underwent clinical examination, bilateral mammography, and bilateral breast and axillary ultrasound. In selected cases, MRI and PET–CT were performed. Suspicious findings were histologically confirmed via VAB or CNB. In patients with suspicious axillary lymph nodes, VAB or CNB was also performed to rule out metastatic involvement prior to surgery. Surgical management consisted of either mastectomy or breast-conserving surgery, combined with SLNB in all included cases.

### 2.6. Sentinel Lymph Node Mapping and Biopsy

Sentinel lymph node mapping was performed using a radioactive tracer (technetium-99m, Tc-99m). Peritumoral or periareolar injection of Tc-99m nanocolloid was administered within 24 h prior to surgery. Intraoperatively, a handheld gamma probe was used to detect radioactive uptake and guide sentinel lymph node (SLN) excision [8].

Excised SLNs were assessed intraoperatively using the One-Step Nucleic Acid Amplification (OSNA) assay, a molecular diagnostic technique that quantifies cytokeratin 19 (CK19) mRNA copy number in homogenized lymph node tissue. Based on OSNA results, intraoperative decisions regarding further axillary surgery (e.g., axillary lymph node dissection) were made following institutional protocols. Final histopathological confirmation was also performed postoperatively [9].

### 2.7. Breast Cancer in Older Women: Clinical and Therapeutic Considerations

Breast cancer is a biologically and clinically heterogeneous disease, comprising various subtypes with distinct prognostic implications at molecular, biological, and clinical levels. In early-stage disease, systemic adjuvant therapy is commonly employed to reduce the risk of locoregional and systemic recurrence, as well as disease-related mortality [10].

Therapeutic decisions are guided by a combination of clinical factors (e.g., age, comorbidities) and pathological parameters (e.g., tumor size, nodal status, histological grade, Ki67 proliferation index, hormone receptor [HR] status, and HER2 status). Moreover, multigene expression assays provide prognostic and predictive information that surpasses traditional clinical and pathological factors and may assist in tailoring treatment strategies [10].

#### Therapeutic Approach in Older Women

In elderly patients, individualized treatment planning is essential, considering functional status, life expectancy, and patient preferences. The primary objective is to achieve effective disease control without compromising overall survival or quality of life [11].

Radiotherapy is generally well tolerated in older women and has demonstrated safety across all age groups [12].

A significant proportion of breast cancers in older women are hormone receptor-positive, with prevalence increasing with age—reaching approximately 91% at 85 years [13].

Given its favorable safety profile and proven efficacy, endocrine therapy is recommended in HR-positive breast cancer regardless of age, tumor size, or nodal involvement, as it significantly reduces the risk of recurrence and breast cancer-specific mortality [14].

Chemotherapy may be an appropriate option for selected elderly patients, particularly those with

- Good performance status and minimal comorbidities,
- An estimated life expectancy of ≥5 years,
- HR-negative tumors with nodal involvement,
- High-risk node-negative disease.

Evidence supports that the survival benefits associated with adjuvant chemotherapy—both in terms of overall survival (OS) and progression-free survival (PFS)—are comparable to those seen in younger populations [15].

### 2.8. Variables Collected

The following variables were collected:

- Demographic and clinical data: age, sex, comorbidities, ASA physical status classification.
- Tumor characteristics: histological type, tumor grade, molecular subtype.
- Axillary assessment and previous history: imaging method, prior diagnosis of breast cancer.
- Surgical details: type of breast surgery, SLNB (number of nodes removed and involved), axillary lymph node dissection, pathological nodal staging, and outpatient surgery status.
- Adjuvant treatment: radiotherapy (breast and axilla), hormone therapy, and chemotherapy.
- Oncological outcomes: local (breast and axillary) recurrence and overall mortality.

### 2.9. Statistical Analysis

Continuous variables were expressed as mean and standard deviation (SD) if normally distributed, or as median and interquartile range (IQR) if non-normally distributed. Categorical variables were described using absolute and relative frequencies (n and percentage). Group comparisons were not conducted, as no control or comparison group was included in the study design.

Violin plots were used to visually display the distribution of continuous variables, including the number of sentinel lymph nodes removed and the number of metastatic nodes. These plots combine a boxplot with a kernel density estimation, allowing for a more detailed depiction of the underlying distribution and variability of the data.

To evaluate the diagnostic performance of different preoperative imaging modalities (e.g., ultrasound, MRI, PET–CT) in predicting axillary involvement, receiver operating characteristic (ROC) curves were constructed. The area under the ROC curve (AUC) was calculated to quantify the overall discriminative ability of each modality. Sensitivity, specificity, positive predictive value (PPV), and negative predictive value (NPV) were also reported.

Additionally, a sensitivity analysis was performed to assess the robustness of findings related to false-negative axillary assessments. This analysis examined whether diagnostic accuracy varied according to tumor size, molecular subtype, or surgical approach (mastectomy vs. breast-conserving surgery). Subgroup analyses were visualized using stratified ROC curves and AUC comparisons.

All statistical analyses were conducted using RStudio (version 4.3.1) as the integrated development environment.

## 3. Results

A total of 149 patients with breast cancer (BC) and clinically negative axillae were included. The mean age was 77.2 (5.2) years. The vast majority were women (99.3%; n = 148). Regarding ASA classification, 77.2% (n = 115) of the patients were classified as ASA II. A history of prior breast cancer was recorded in 12.8% (n = 19) of patients (Table 1).

The most frequent histological type was invasive carcinoma of no special type (NST) (n = 87, 58.4%). In terms of histological grade, 70.5% of tumors (n = 105) were classified as grade II. Regarding tumor size, 45.6% (n = 68) measured between 10 mm and 20 mm (T1c), followed by 24.8% (n = 37) measuring between 20 mm and 50 mm (T2). The predominant molecular subtype was luminal (85.9%; n = 128) (Table 2). In our cohort, lymphovascular invasion was identified in 15% of breast cancer cases. In our study, surgical margins were negative (R0) in 98.2% of cases, while 1.8% showed close or positive margins requiring further treatment.

**Figure 1:**
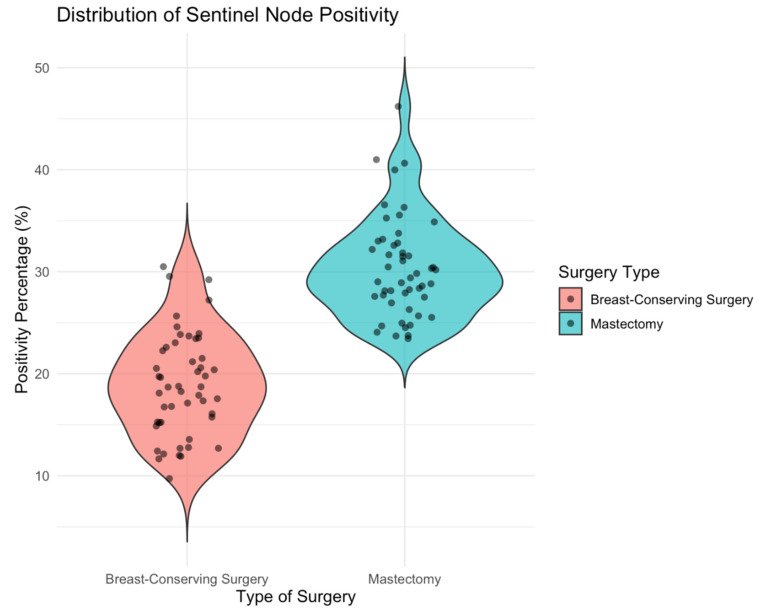
Violin plot illustrating the distribution of sentinel lymph node positivity.

As for surgical approach, 98 patients (65.8%) underwent breast-conserving surgery, while 51 (34.2%) underwent mastectomy. Sentinel lymph node positivity was observed in 23.5% (n = 35) of patients, of whom 20 (57.1%) had macrometastases. Axillary lymph node dissection (ALND) was performed in 10 patients (6.7%) (Figure 1). No cases of breast or axillary recurrence were identified.

Regarding adjuvant treatment, 68.5% (n = 102) received breast radiotherapy, and 17.5% (n = 26) received axillary radiotherapy. Hormone therapy was administered to 134 patients (89.9%), while only 17 (11.4%) received adjuvant chemotherapy (Table 3).

The overall mortality rate was 1.3% (n = 2), and 87.9% (n = 131) were managed on an outpatient basis without requiring hospitalization.

### 3.1. Analysis by Type of Surgery

#### 3.1.1. Mastectomy Group

**Figure 2:**
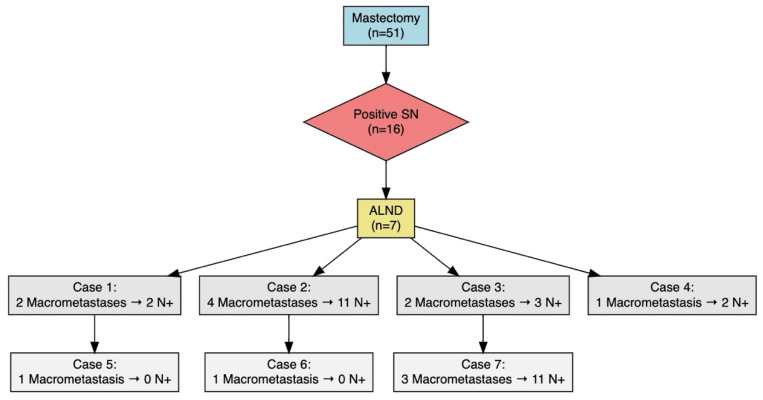
Flowchart showing the distribution of axillary dissection outcomes in mastectomy patients with positive sentinel lymph node biopsy meeting criteria for axillary dissection. SN: sentinel node, ALND: axillary lymph node dissection.

Sentinel lymph node positivity was observed in 16 patients (31.4%). Of these, nine had micrometastases and did not undergo ALND. Among the remaining seven patients, the distribution of sentinel lymph node findings was as follows (Figure 2):

- Three patients with one macrometastasis (two of whom had no additional positive nodes on ALND).
- Four patients with two or more macrometastases, all of whom had additional positive lymph nodes on ALND. In this subgroup, 100% of patients who underwent ALND had further nodal involvement.

Notably, all patients in the mastectomy group had tumors smaller than T3 (Table 4).

#### 3.1.2. Breast-Conserving Surgery Group

**Figure 3:**
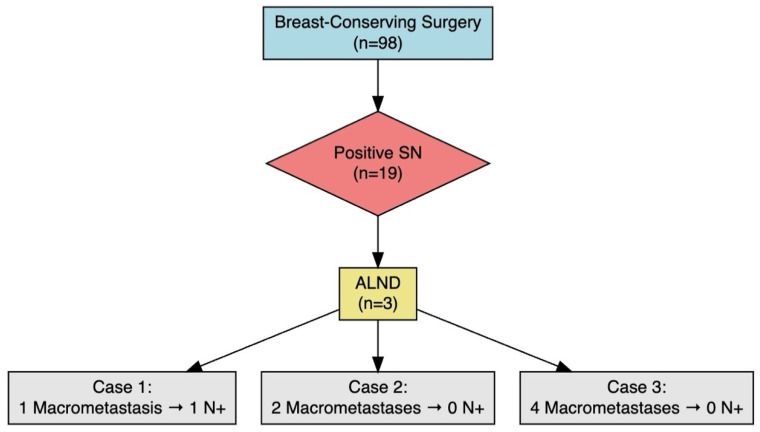
Flowchart showing the distribution of axillary dissection outcomes in patients undergoing breast-conserving surgery with positive sentinel lymph node biopsy meeting criteria for axillary dissection. SN: sentinel node, ALND: axillary lymph node dissection.

Sentinel lymph node positivity was observed in 19 cases (19.4%). ALND was performed in three patients (15.8%), while the remainder did not undergo further dissection, in accordance with the ACOSOG Z0011 trial criteria [8,9,10,11,12,13,14,15,16]. Of the patients who underwent ALND, only one had an additional positive node. This patient had a macrometastatic sentinel node (total tumor load: 1,200,000 copies) (Figure 3).

Although the rate of positive nodes was higher in the mastectomy group compared to the breast-conserving group, the difference in sentinel lymph node positivity between the two groups was not statistically significant (*p* = 0.1517).

#### 3.1.3. Sensitivity and Specificity Analysis of Sentinel Node According to Age

**Figure 4:**
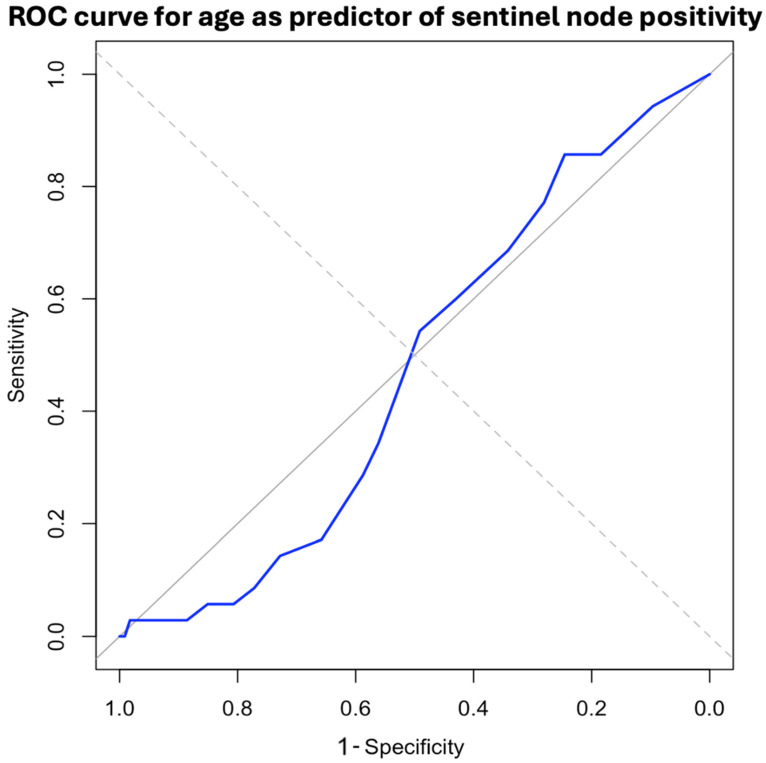
ROC curve for age as predictor of sentinel node positivity.

A receiver operating characteristic (ROC) curve (Figure 4) was constructed to evaluate the sensitivity and specificity of sentinel lymph node positivity in relation to age. It was observed that, from the age of 76 onwards, both sensitivity and specificity decreased significantly, reaching values of 57.14% and 53.95%, respectively (Table 5).

## 4. Discussion

The surgical approach to treating breast cancer (BC) in women over the age of 70—particularly those with luminal tumors and clinically negative axillae—has shifted towards a de-escalation strategy. This trend is supported by growing evidence indicating that invasive procedures such as sentinel lymph node biopsy (SLNB) may be unnecessary in selected cases. This consideration is especially relevant in older patients and is becoming increasingly accepted, particularly beyond the age of 80 [17,18]. In our series, the nodal involvement rate was 23.5%. Studies such as that by Panadés et al. [19] report that women over 70 often present with larger, more advanced tumors, resulting in less frequent axillary surgical management—SLNB was performed in only 69.1% of cases. This highlights the importance of personalizing treatment strategies based on comorbidities and life expectancy, without compromising disease control [19].

One of the most notable findings of our study was the marked decrease in the sensitivity (57.1%) and specificity (54.0%) of SLNB in patients over the age of 76—a phenomenon not yet widely reported in the literature. This reduced diagnostic performance aligns with increasing interest in omitting axillary procedures in the elderly. In a large retrospective analysis, Minami et al. [20] found that 50.9% of women aged 70 or older with luminal tumors received a de-escalated treatment, omitting axillary surgery and/or radiotherapy. Importantly, 35.3% of the variation in this practice was attributable to regional factors, such as institutional protocols and clinical preferences, while only 2.8% was explained by individual patient characteristics [21]. Similarly, Wang et al. [22], in a qualitative study, observed that 73% of women interviewed would prefer to omit radiotherapy if it did not improve survival, while 40% opted for SLNB, considering it low-risk and offering them “peace of mind” [23]. These findings underscore the need to individualize therapeutic decisions for older women, taking into account not only the real diagnostic value of SLNB but also patient preferences, comorbidities, and regional context.

When comparing types of surgery, we observed a 31.4% rate of nodal positivity in mastectomy cases versus 19.4% in breast-conserving surgery, though the difference was not statistically significant (*p* = 0.1517). Previous research has also shown higher rates of nodal positivity among patients undergoing mastectomy, likely reflecting a selection bias for larger or more aggressive tumors [6,24,25].

In patients with two or more macrometastases, all (100%) had additional nodal involvement on axillary lymph node dissection, reinforcing the conclusions of the ACOSOG Z0011 trial, which suggested that ALND should be reserved for patients with high nodal burden [5,16]. Studies by Chagpar et al. [26] and Esposito et al. [27] also support omitting ALND in patients with micrometastases or minimal involvement, given the lack of significant differences in survival or locoregional control [26,27,28].

At the systemic level, 89.9% of our patients received hormone therapy, while only 11.4% underwent chemotherapy. This trend aligns with current evidence suggesting that nodal status has limited impact on chemotherapy decisions for older women with luminal BC [29]. Treatment decisions should be guided more by tumor biology and functional status than by nodal involvement [30,31,32]. Axillary radiotherapy was administered in 17.5% of cases, with no locoregional recurrences reported. The PRIME II trial demonstrated that omitting radiotherapy is safe in older women with hormone receptor-positive tumors and negative margins receiving adjuvant endocrine therapy [33,34].

Our data also support the functional safety of avoiding axillary surgery: no clinically significant cases of lymphoedema or sensory disturbance were reported. Given that advanced age increases the risk of postoperative complications, surgical de-escalation offers a clear functional benefit [17,35].

From an organizational perspective, 87.92% of patients were managed on an outpatient basis. McEvoy et al. [36] highlight the benefits of reduced hospital stay and cost savings from omitting SLNB, particularly in high-volume surgical centers. From an economic standpoint, the cost-effectiveness of SLNB in elderly women with negative axillae is questionable. Hrebinko et al. [37] conducted a cost-effectiveness analysis showing that omitting SLNB not only reduces healthcare costs but also improves quality-adjusted life years (QALYs) in women over 70 with luminal BC and no clinical evidence of nodal involvement—supporting the notion that conservative surgical selection is not only safe and effective, but also more efficient in public health terms [37].

Minami et al. [20] also reported substantial institutional variability in SLNB practices, linked to resource availability and surgical team expertise [20,21,38]. Despite the recommendations of scientific societies such as the Society of Surgical Oncology and Choosing Wisely, systematic implementation remains challenging. In many centers, SLNB continues to be performed by default, without an individualized risk–benefit assessment [3,24].

Nevertheless, patient acceptance is high when adequate information is provided. The literature shows that most older women would prefer to avoid axillary surgery when it offers no clinical benefit, emphasizing the importance of shared decision-making [29].

Alamoodi et al. [39] stress that de-escalation not only improves quality of life, but may also reduce psychological complications by avoiding interventions that heighten postoperative anxiety and fear. Carleton et al. [40] found that omitting SLNB has no impact on overall or disease-free survival and allows therapeutic strategies to focus on more relevant systemic treatments.

Chagpar et al. [26] further highlighted the evolution of surgical practice over the past fifteen years, showing that omission of SLNB is now common in academic centers with multidisciplinary teams. Jatoi et al. [33] provide a critical review of the history, indications, and future of SLNB, calling for a reassessment of its value in older women with early-stage BC.

Several studies also report that the rate of clinically significant nodal involvement is very low in older women, supporting the omission of SLNB in this group. Even in cases of micrometastases, clinical progression is uncommon in women receiving adequate hormonal therapy, while the risk of lymphoedema increases with unnecessary ALND [41]. In short, survival benefits are limited in comparison to the negative impact on quality of life [42].

Despite growing expert consensus on the need to de-escalate treatment in older women with luminal BC, few prospective studies have focused on this group. The study by Chung et al. [43] provides compelling data: among 125 women aged over 65 with ER+ BC treated with breast-conserving surgery without SLNB, the three-year axillary recurrence rate was 1.6% and the overall survival rate was 94.8%, even though only 48% were still on endocrine therapy after two years. These findings offer robust evidence supporting omission of SLNB in this population.

Beyond clinical outcomes, the debate has evolved through contributions from molecular biology. Reimer et al. [44] point out that the prognostic value of nodal status in clinically node-negative patients is now surpassed by information derived from tumor subtype and genomic profiling [45]. This challenges the utility of SLNB as a decision-making tool for adjuvant therapy. Similarly, the meta-analysis by Kell et al. [46] concluded that, while SLNB is less invasive than ALND, its clinical benefit is questionable when nodal risk is low and systemic therapy is already indicated based on other tumor characteristics.

In this context, Lewis et al. [47] demonstrated excellent survival outcomes with tamoxifen and toremifene in postmenopausal women with early-stage luminal BC, reinforcing the importance of endocrine therapy. Additionally, tumor size below 1 cm remains one of the best predictors of nodal negativity, supporting the decision to omit SLNB in patients with small tumors [25].

Data on women over the age of 80 provide an even more radical yet meaningful perspective. In the study by Acosta et al. [18], none of the 36 patients who did not undergo axillary intervention developed axillary recurrence. Disease-free survival reached 81.0% and overall survival 57% at five years, despite most patients receiving only breast surgery and endocrine therapy.

The recent INSEMA trial prospectively examined the omission of SLNB in women with early-stage BC (cN0) undergoing breast-conserving surgery plus radiotherapy. Results showed no significant differences in disease-free or locoregional recurrence, strongly supporting reduced axillary surgical management in appropriately selected patients [45].

Similarly, Li X [22] observed that practice in many European hospitals remains driven by protocol rather than clinical judgement, despite mounting evidence favoring less interventionist approaches. In patients with imaging-negative axillae, omitting SLNB does not increase locoregional recurrence or reduce survival, particularly in women over 70 with luminal tumors and low Ki-67 [48]. Williams et al. [49] concluded that eliminating intraoperative tools such as frozen section analysis reduces overtreatment and streamlines surgery without compromising oncological control.

Hu X et al. [50] proposed a preoperative nomogram to predict nodal involvement in BC patients. This model showed good discriminatory power and could help avoid SLNB in older women with low-risk clinical features, thus contributing to a more personalized surgical de-escalation.

This study presents several limitations that should be acknowledged. Its retrospective design may introduce selection and information biases inherent to medical record review. Although the data were extracted from a prospectively maintained institutional registry, unmeasured confounders cannot be excluded. Moreover, the study included only patients who underwent SLNB, which precludes a direct comparison with patients in whom the procedure was omitted. As such, it is not possible to assess the actual impact of omitting SLNB on oncological outcomes in this population. In addition, the short follow-up duration limits the ability to assess long-term outcomes. Although no axillary or breast recurrences were observed in the study cohort, the median follow-up of less than two years is insufficient to draw meaningful conclusions regarding long-term oncological outcomes. Extended follow-up is essential to adequately assess recurrence rates in this population. Lastly, the study was conducted at a single tertiary center, which may limit the generalizability of the findings to other clinical settings or healthcare systems. Furthermore, in light of the modest diagnostic performance observed in patients over 76 years, future studies should explore complementary or alternative strategies to improve the accuracy of axillary staging in this population.

In summary, our findings support the growing body of evidence indicating that omission of SLNB is safe, efficient, and clinically appropriate for women over 70 with luminal tumors, negative imaging of the axilla, and appropriate systemic treatment. Personalized approaches, shared decision-making, and proper clinical selection are key to advancing towards less invasive and more value-centred surgery.

## 5. Conclusions

Our research highlights a growing agreement that skipping sentinel lymph node biopsy is a safe and sensible choice for women aged 70 and older who have luminal breast cancer and negative axillary imaging. The low rates of axillary recurrence, the minimal need for axillary dissection, and the ease of managing patients on an outpatient basis all support a shift towards a more conservative strategy for this group. Additionally, the decreased effectiveness of SLNB in patients over 76, along with the limited influence of nodal status on systemic therapy choices, raises questions about the routine use of axillary staging in these cases.

This study was specifically designed to assess whether SLNB omission could be a viable strategy in our local setting, aiming to evaluate its true clinical utility. Based on our findings, a progressive change in clinical practice is currently underway at our institution, moving toward the omission of SLNB in this age group when appropriate clinical and radiological criteria are met. This transition reflects a broader shift toward personalized, less invasive, and value-based surgical management in elderly breast cancer patients.

## Figures and Tables

**Table 1:** Clinical and demographic features.

			Mastectomy (%)	Breast-Conserving Surgery (%)	Total (%)
n			51 (34.2)	98 (65.7)	149
Age	Mean age (SD) (years)		78.5 (5.8)	76.5 (4.8)	77.2 (5.2)
	95% CI range (years)		76.9–80.2	75.5–77.5	76.34–78.0
Sex	Female		50 (98.0)	98 (100.0)	148 (99.3)
	Male		1 (2.0)	0 (0.0)	1 (0.7)
ASA	I		1 (2.0)	0 (0.0)	1 (0.7)
	II		36 (70.6)	79 (80.6)	115 (77.2)
	III		13 (25.5)	19 (19.4)	32 (21.5)
	IV		1 (2.0)	0 (0.0)	1 (0.7)
	V		0 (0.0)	0 (0.0)	0 (0.0)
Breast laterality	Right		22 (43.1)	57 (58.2)	79 (53.0)
	Left		29 (56.9)	41 (41.8)	70 (47.0)
Previous breast neoplasm			6 (11.8)	13 (13.7)	19 (12.8)
	Breast laterality	Ipsilateral	1 (16.7)	1 (7.7)	2 (10.5)
		Contralateral	5 (83.3)	12 (92.3)	17 (89.5)
	Molecular type	Same	3 (50.0)	5 (38.5)	8 (42.1)
		Different	2 (33.3)	7 (53.9)	9 (47.4)
		Not available	1 (16.7)	1 (7.7)	2 (10.5)
Imaging diagnosis	Ultrasound		51 (100.0)	98 (100.0)	149 (100.0)
	MRI		11 (21.6)	19 (19.4)	30 (20.1)
	PET–CT		0 (0.0)	1 (1.0)	1 (0.7)

Categorical variables are presented as counts and percentages, n (%). SD: standard deviation, CI: confidence interval.

**Table 2:** Anatomopathological features of the surgical specimen.

			Mastectomy (%)	Breast-Conserving Surgery (%)	Total (%)
Histological type	Invasive carcinoma of no special type		24 (47.1)	63 (64.3)	87 (58.4)
	Invasive lobular carcinoma		16 (31.4)	14 (14.3)	30 (20.1)
	Invasive ductal carcinoma		2 (3.9)	3 (3.1)	5 (3.4)
	Mucinous carcinoma		6 (11.8)	4 (4.1)	10 (6.7)
	Solid papillary carcinoma		1 (2.0)	3 (3.1)	4 (2.7)
	Invasive micropapillary carcinoma		0 (0.0)	5 (5.1)	5 (3.4)
	Mixed invasive carcinoma		2 (3.9)	0 (0.0)	2 (1.3)
	Invasive papillary carcinoma		0 (0.0)	1 (1.0)	1 (0.7)
	Others types		0 (0.0)	5 (5.1)	5 (3.4)
T stage	T1		25 (49.0)	82 (83.7)	107 (71.8)
	T2		23 (45.1)	14 (14.3)	37 (24.8)
	T3		3 (5.9)	2 (2.0)	5 (3.4)
	T4		0 (0.0)	0 (0.0)	0 (0.0)
Histological grade	I		8 (15.7)	26 (26.5)	34 (22.8)
	II		40 (78.4)	65 (66.3)	105 (70.5)
	III		3 (5.9)	7 (7.1)	10 (6.7)
Molecular type	Luminal A		15 (29.4)	52 (53.1)	67 (45.0)
	Luminal B		27 (52.9)	34 (34.7)	61 (40.9)
	Luminal B-Her2+		3 (5.9)	2 (2.0)	5 (3.4)
	Her2+		3 (5.9)	3 (3.1)	6 (4.0)
	Triple-negative		3 (5.9)	7 (7.1)	10 (6.7)
SLNB	Positive		16 (31.4)	19 (19.4)	35 (23.5)
	Negative		35 (68.6)	79 (80.6)	114 (76.5)
	SLNB features	>1 micrometastases	8 (50.0)	7 (36.8)	15 (42.9)
		≥1 macrometastases	7 (43.8)	8 (42.1)	20 (57.1)
		Macrometastases + micrometastases	1 (6.3)	4 (21.1)	5 (27.3)
ALND			7 (13.7)	3 (3.1)	10 (6.7)
	ALND features	pN0	35 (66.7)	77 (77.6)	112 (75.2)
		pN1	13 (25.5)	20 (20.4)	33 (22.2)
		pN2	1 (2.0)	1 (1.0)	2 (1.3)
		pN3	2 (3.9)	0 (0.0)	2 (1.3)

Categorical variables are presented as counts and percentages, n (%). ALND: axillary lymph node dissection (ALND), SLNB: sentinel lymph node biopsy, T: tumor.

**Table 3:** Adjuvant treatment.

			Mastectomy (%)	Breast-Conserving Surgery (%)	Total (%)
Breast adjuvant radiotherapy			16 (37.2)	86 (87.8)	102 (68.5)
Axillary adjuvant radiotherapy			9 (20.9)	17 (17.4)	26 (17.5)
	Irradiated lymph node levels	I	6 (66.7)	15 (88.2)	20 (76.9)
		II	6 (66.7)	15 (88.2)	20 (76.9)
		III	7 (77.8)	9 (52.9)	13 (50.0)
		IV	1 (11.1)	6 (35.3)	7 (26.9)
		V	0 (0.00)	1 (5.9)	1 (3.9)
Breast adjuvant hormonotherapy			45 (88.2)	89 (90.8)	134 (89.9)
Breast adjuvant chemotherapy			8 (15.7)	9 (7.5)	17 (11.4)

Categorical variables are presented as counts and percentages, n (%).

**Table 4:** Cross-table of positive sentinel node with criteria for axillary lymph node dissection and positive nodes in axillary dissection.

Mastectomy:						
	ALND					
	N+	0	1	2	3	11
SLNB with criteria for ALND	1	2		1		
	2			1	1	
	3					1
	4					1
**Breast-conserving surgery:**						
	ALND					
	N+	0	1	2	3	11
SLNB with criteria for ALND	1		1			
	2	1				
	3					
	4	1				

SLNB: sentinel lymph node biopsy, ALND: axillary lymph node dissection.

**Table 5:** Study of sensitivity and specificity of SLNB according to age.

Age (years)	Sensitivity	Specificity
70	0.9429	0.9035
71	0.9000	0.8596
72	0.8571	0.7851
73	0.8143	0.7368
74	0.7286	0.6886
75	0.6429	0.6140
76	0.5714	0.5395
77	0.4429	0.4737
78	0.3143	0.4254
79	0.2286	0.3772
80	0.1571	0.3070
81	0.1143	0.2500
82	0.0714	0.2105
83	0.0571	0.1711
84	0.0429	0.1316
85	0.0286	0.0965
86	0.0286	0.0702
87	0.0286	0.0526
88	0.0286	0.0395
89	0.0286	0.0307
90	0.0286	0.0219
91	0.0214	0.0154
92	0.0071	0.0110
93	0.0000	0.0088

## Data Availability

The data presented in this study are not publicly available due to ethical and privacy restrictions, in accordance with the approval conditions established by the Clinical Research Ethics Committee of Hospital Clínico Universitario of Valencia.
